# Acute regadenoson stress prefusion testing using cardiac MRI is a valuable test of high predictive value for risk stratification of COPD-patients with NSTEM

**DOI:** 10.1186/1532-429X-18-S1-P105

**Published:** 2016-01-27

**Authors:** Felix T Range, Florian Bönner, Sebastian M Haberkorn, Dirk Dinjus, Stefanie Keymel, Malte Kelm, Mirja Neizel-Wittke

**Affiliations:** grid.14778.3d0000000089227789Dept. of Cardiology, Pneumology and Angiology, University hospital of Düsseldorf, Neuss, Germany

## Background

The diagnostic dilemma of patients suffering from chronic obstructive pulmonary disease (COPD) presenting with symptoms equivalent to an acute coronary syndrome (ACS) and slightly elevated high sensitive troponine in the observational range (hsTnT 14-53 ng/l) is complex. Prognostic impact of elevated hsTnT demand further investigation. Comorbidities of COPD and coronary artery disease are frequent, risk factors congruent. The acute setting demands a safe and easily conductible test to identify coronary problems reliably. Echocardiographic imaging quality is often low due to emphysema. Adenosine is contraindicated due to its bronchospasmic effect. The new vasodilatator regadenoson does not bear this limitation.

This study aims to evaluate feasibility and prognostic power of regadenoson stress cMRI in the above described population.

## Methods

Twenty-two patients (70 ± 13 yo) with COPD GOLD grade I-III presenting with ACS and elevated hsTnT in the observational range (23 ± 9 ng/l) were enrolled. Prior to coronary catheterization, they underwent cMRI with regadenoson-stress (400 μg i.v. bolus). Scans were conducted on a Philips Achieva 1.5 T MRI, including CINE-sequences, T2-imaging, late enhancement imaging, pulmonary angiography as well as rest and stress myocardial perfusion measurements. All Patients received pulmonary function testing before and after regadenoson. All scans were conducted within 48 hours after presentation.

## Results

No adverse effect of regadenoson was observed. Pulmonary function testing revealed a slight but not significant amelioration after regadenoson (FEV1 pre and post MRI: 67% and 73%, p = ns).

Quality of the scans remained unhampered by the emergency management. All scans were conducted within 48 hours after presentation, also allowing consecutive coronary catheterization within this time frame. Regadenoson elevated heart rate and rate-pressure-product significantly by 30%. In eight patients, stress cMRI detected myocardial ischemia. In all these patients, coronary catheterization revealed relevant coronary stenoses necessitating intervention. In all cases, the respective vessel was correctly predicted by cMRI. One patient could not be intervened due to a very small diameter of the target vessel. Whenever cMRI detected no myocardial ischemia, coronary catheterization revealed no coronary stenosis. We found positive and negative predictive values of cMRI with regadenoson-stress at 100% in our population.

## Conclusions

Cardiac stress MRI proved safe, feasible in a limited timeframe and of high predictive accuracy in our study. Regadenoson did not deteriorate pulmonary function. The quality of cMRI scans and analysis did not suffer from the emergency setting and positive and negative predictive values were 100% when determining the need for coronary revascularisation and predicting the target vessel.

This method is ready to be implemented in a differentiated management of patients with COPD and slightly elevated hsTnT.

